# Spatial Covariance Reconstructive (SCORE) Super-Resolution Fluorescence Microscopy

**DOI:** 10.1371/journal.pone.0094807

**Published:** 2014-04-30

**Authors:** Yi Deng, Mingzhai Sun, Pei-Hui Lin, Jianjie Ma, Joshua W. Shaevitz

**Affiliations:** 1 Department of Physics, Princeton University, Princeton, New Jersey, United States of America; 2 Lewis-Sigler Institute for Integrative Genomics, Princeton University, Princeton, New Jersey, United States of America; 3 Department of Physiology and Biophysics, Robert Wood Johnson Medical School, Piscataway, New Jersey, United States of America; University of California, Berkeley, United States of America

## Abstract

Super-resolution fluorescence microscopy has become a powerful tool to resolve structural information that is not accessible to traditional diffraction-limited imaging techniques such as confocal microscopy. Stochastic optical reconstruction microscopy (STORM) and photoactivation localization microscopy (PALM) are promising super-resolution techniques due to their relative ease of implementation and instrumentation on standard microscopes. However, the application of STORM is critically limited by its long sampling time. Several recent works have been focused on improving the STORM imaging speed by making use of the information from emitters with overlapping point spread functions (PSF). In this work, we present a fast and efficient algorithm that takes into account the blinking statistics of independent fluorescence emitters. We achieve sub-diffraction lateral resolution of 100 nm from 5 to 7 seconds of imaging. Our method is insensitive to background and can be applied to different types of fluorescence sources, including but not limited to the organic dyes and quantum dots that we demonstrate in this work.

## Introduction

The information provided by fluorescence imaging is essentially limited by the resolution, defined as the finest structure that the imaging technique is able to resolve. The resolution is critically limited by the diffraction of the objective aperture, which places physical challenges to obtaining information from the acquired images. Recently, several novel imaging techniques has been developed aiming to break the diffraction limit, namely super-resolution imaging. These techniques can be categorized into three different types. The first type utilizes a spatially or temporally engineered illumination pattern to either reduce the volume of the detected target in a controllable way, as in stimulated emission depletion (STED) microscopy [1]–[3] which uses reversible, saturable optical fluorescence transitions (RESOLFT) [4], or encodes high-frequency spatial information into low-frequency signals such as in saturated structural illumination microscopy (SSIM) where angular or temporal responses of the fluorophore are used as spatial information carriers [5]. These types of imaging techniques usually require much higher excitation or switching power than conventional epi- or confocal fluorescence microscopy, and may cause phototoxicity and photodamage in certain biological systems. The instrumentation complexity and cost are considerations that also limit the applicability of these methods.

The second group of methods resolves the distribution of fluorescent sources based on single particle localization. Stochastic optical reconstruction microscopy (STORM) [6] and photo-activated localization microscopy (PALM) [7], [8] achieve a resolution of 10 nm by sequentially activate single emitters and precisely localizing them to better than the diffraction limit [9]. However, the limitation of STORM/PALM lies in the fact that the resolving ability is based critically on the spatial and temporal separation of emitters, thus the number of emitters resolved in each frame is small compared with the required amount for good image reconstruction. A reconstructed image typically requires 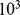 to 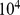 images, or minutes of imaging time [10], for reasonable quality. Recently, sub-minute acquisition times have been achieved for relative large structures with a similar number of frames [11]. The time required for imaging disqualifies the use of these approaches in most live-cell imaging applications. Aiming to obtain high information density by looking at more densely distributed bright emitters, Holden *et al.* used a software package from astronomy research to separate closely located Gaussians in a technique called DAOSTORM [12]. Compressed sensing STORM (CSSTORM) and deconSTORM also address the overlapping PSF problem and are able to work with slightly a overlapping density of active emitters [13], [14]. Higher emitter density still remains challenging for these essentially single emitter localization based methods. Furthermore, the computational costs of these sophisticated algorithms often outweigh the improvements of the quality, making them less desirable as routine super-resolution imaging methods.

Instead of resolving the localization of emitters based on single switching events, a third group of approaches extracts localization information from the statistics of pixel intensity fluctuations. In these approaches, overlapping emitters are actually preferred since a higher density of provides higher intensity variance. Many different kinds of emitters including fluorescent proteins, organic dyes [10] and nano crystals [15], [16] have particular temporal intensity fluctuation patterns, or fluorescence intermittency (FI). Based on the independence of the emitter intensity fluctuations from each molecule, super-resolution optical fluctuation imaging (SOFI) was developed to effectively reduced the PSF width by taking the simple temporal cumulant of the pixel intensities [17]. SOFI was later improved by including spatial cross-cumulants of the intensity fluctuations [18]. Following these same principles, Lidke *et al.* resolved the localization of densely distributed individual quantum dots from the spatial covariance of the pixel intensity fluctuations using independent component analysis (ICA) [19], [20]. This method was shown to be applicable to a small number of sources but becomes intractable if the number of emitter reaches 10 or more, and thus is not applicable to resolving continuous structures. A realistic model that optimizes the location and blinking of emitters was built by Cox *et al.* to yield super-resolution images at a greatly improved temporal resolution of seconds [21]. The computational complexity of this global optimization is however extremely high, making it difficult to implement. These statistical methods reach a resolution of ∼100 nm that can not match the single particle localization techniques, but with a far smaller number number of time frames (typically in the hundreds), and thus better temporal resolution.

In this work, we introduce a new image reconstruction algorithm belonging to the third category named spatial covariance reconstructive (SCORE) algorithm. We start from the fluorescent intensity covariance between pixels of a stack of images, combine the prior knowledge of the shape of the PSF to map the distribution probability of individual emitters. Our method builds upon the common advantages of the statistical approaches: insensitivity to background, no upper limit to the fluorescent emitter on-time, far higher information density per frame, low requirements on the number of frames, and ease of implementation from standard epi-fluorescence instruments. Through principal component data compression, we only consider components that have significant contribution to the variance of pixel intensities, and thus this method reduces image noise and computational cost. We quantitatively compare the results of STORM and SCORE using simulated data, where the quality is defined as the Kullback-Leibler divergence from the known ground truth to the resulted images. As demonstrations, we applied SCORE to images of microtubules in HeLa cells labeled with either organic dyes and quantum dots. In both cases, we are able to achieve sub-diffraction resolution of better than 135 nm and 90 nm, respectively, within a few seconds of imaging. The resolution is limited by our labeling quality, and better imaging resolution can be obtained by using fluorophores that have more robust intensity fluctuations.

## Results

We consider *M* fluorescent emitters located at positions 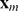 in a 2D plane. Here we do not place constraints on the spatial distribution of 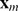, so that the emitters can be arbitrarily dense. Each emitter undergoes statistically independent fluctuations in intensity over time governed by the photochemistry of the particular molecules used. We record a sequence of *T* images of these emitters, whose images are governed by the instantaneous intensity of each emitter 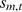 where 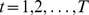. The independence condition of different emitters requires that 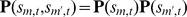 for 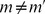. Each emitter is imaged as a diffraction limited spot described by the PSF. The size of the PSF depends on the imaging wavelength, the numerical aperture of the objective and the axial distance of the emitter from the focal point. In our model, we assume that the PSFs are spatially homogeneous. Let the individual PSF be given by 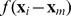 which describes the the intensity value of the 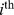 pixel at location 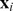 of a 2D PSF centered at the 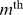 source location 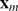. The temporal sequence of image pixel intensities in the absence of noise is 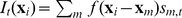. The convolution can be rewritten in vector notation 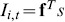, where 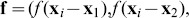 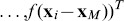, and 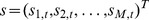.

**Figure 1:**
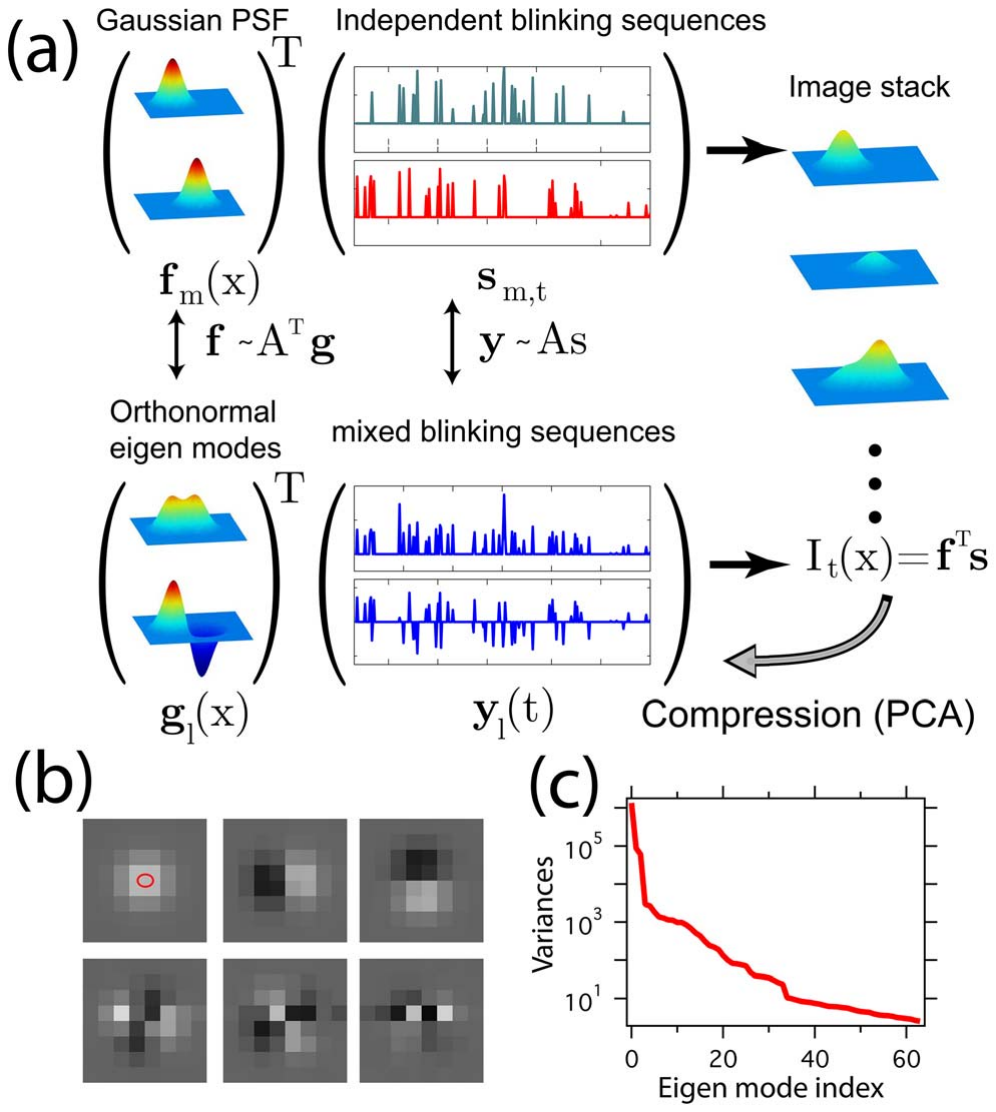
The SCORE technique illustrated using simulated data. (a) Two emitters produce a Gaussian shaped point spread function 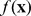, associated with a temporal blinking sequence 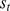 that is independent one another. The product of the spatial profiles and temporal sequences is the observable images, which can be transformed into a set of orthonormal eigen modes 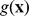 and associated mixed and thus correlated fluctuation sequences 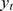. The distribution of the emitters can be found by measuring the distance between a Gaussian point spread function and the subspace span by the eigen modes that have significant variation above noise. (b) First 6 eigen modes of flickering emitters distributed evenly on a sub-diffraction sized ellipse indicated by the red oval. (c) The sorted variances of all the 64 eigenmodes.

The problem of image reconstruction is to find the best estimate for the emitter positions 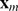 from the observed time series. The PSF 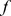 is usually well approximated by a Gaussian function in two-dimensional cases, and 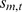 is assumed to follow mutually independent and identical statistics (Fig. 1). The difficulty of globally optimizing the emitter locations and intensity sequences lies in the fact that the number of emitters, 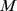, is unknown and the parameter space is extremely high-dimensional. It helps to reduce the dimensionality of the problem by truncating the principal components of the data set above the noise level as suggested in reference [19]. Rather than seeking the solution in terms of the locations, we coarse-grain the set of center locations, 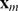, onto a finite mesh grid with points located at 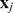, 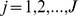, which represents an up-sampled mesh of the camera pixel array 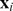. In reconstructing an image, we estimate the number of emitters, 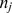, at each grid point.

The principal components can be obtained from the covariance matrix between pixels in a time-series of images
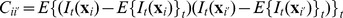
(1)where 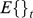 takes the average over time index 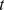. The normalized eigenvectors of 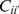 form an orthonormal basis set that explains the variations of pixels in groups, while the corresponding eigenvalues are equal to the variance of the corresponding eigenmode amplitudes. The total number of the eigenmodes, 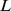, is the same as the number of pixels. We label the corresponding eigenvectors 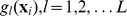, or 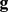 in the vector notation, and the eigenvalues 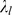.

Dimensional reduction in principal component analysis can be achieved by eliminating those modes with less than a threshold that retains meaningful signals while reducing noise. The eigenvalue spectrum from a typical set of blinking emitter images shows a kink that can be used to separate signal from background noise (Fig. 1(c)). We determine the position of the critical threshold by fitting the variances with a double exponential function, and take the intersection of the two functions at 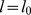 as the threshold between signal and noise (see online supporting material for details). The truncated set of eigenmodes is a compressed representation of the original pixel intensity covariance. Conversely, the individual fluctuating PSFs span a subspace close to the eigen-subspace of the data, but are corrupted by noise and pixelation.

The distance of an arbitrarily placed PSF to the eigen-subspace depends on how close this PSF truly resembles a fluctuating emitter. Quantitatively, we utilize this property to estimate the likelihood of finding a PSF at a particular location 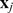 on the refined grid based on the Euclidean distance 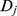 from the PSF to the eigen-subspace 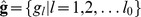. We approximate a PSF centered at 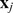 by a Gaussian function with width 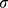: 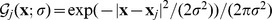, so that the distance is calculated via
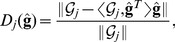
(2)where 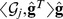 is the projection of the Gaussian-shaped PSF centered at the refined grid position 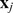 on the compressed subspace 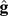, and 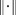 takes the L-2 norm. Note that it is possible to extract emitter localization information on the refined grid 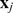 instead of the original image pixel grid, because the localization information is encoded in the shape and variance of the eigen modes.

The relationship between the emitter density and the Euclidean distance is not readily obvious. Therefore, we take an approach that approximates the probability density and then optimizes the distribution so that it resembles the eigen-mode variances obtained from the images. The distance distribution 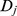 is mapped onto the emitter density by a simple exponential function:
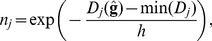
(3)where the maximum value of the image is normalized to 1, and 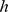 is a parameter that determines the maximum gradient allowed in the reconstructed image. This exponential form is empirically selected. Because of the following optimization steps, the specific form is not critical. Given
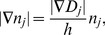
(4)the sharpness parameter, 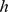, can be implicitly solved numerically. The maximum gradient of the reconstructed image can be selected manually for desired sharpness, or it can be automatically determined without human interference based on the image signal to noise ratio, using the variances of the eigen modes and an empirical relation (see online supporting material for details).

In matrix notation, we define the coordinates of a set of normalized Gaussian functions 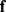 of the eigenvectors 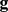 as 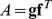. The elements 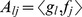 represent the magnitude of the 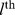 eigenmode of the 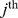 Gaussian function centered at 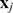. The distance to the subspace, 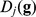, is 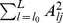 due to the orthogonality of 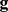. Since 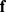 is normalized, we have 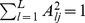, and thus 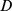 can be written as
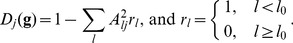
(5)where 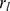 is a set of profiling parameters.

Eq. 5 and Eq. 3 provide a simple and computationally efficient estimate of the emitter number density from the covariance matrix alone using the known shape of the PSF. From the reconstructed image, 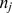, it is straightforward to compute the covariance matrix assuming emitters have independent and identical fluctuation statistics (derived in the supporting materials),
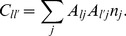
(6)One can then optimize the reconstructed image by comparing the covariance matrix of the reconstructed image in the basis of the eigenvector space 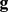 with the covariance of the true images in the same basis. Since the number of pixels in the refined reconstruction is higher than the original image, which has the same size of the covariance matrix, directly optimizing emitter density 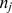 on the refined image is underdetermined. Instead of determining 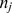, we optimize the profiling parameters, 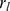, for the significant components 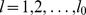 and minimize the weighted error of the covariance of the reconstructed image from the experimental diagonal covariance matrix 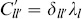,
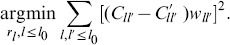
(7)In practice, the weighting matrix is determined via 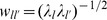. With each set of 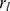, 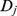 is calculated from Eq. 5 while allowing 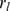 to deviate from 1, and then 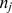 is calculated using Eq. 3 with 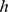 determined using Eq. 4. Eq. 6 is then used to calculate covariance, and finally 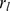 is optimized according to Eq. 7.

The optimized solution is obtained through a gradient descent method, and we name this optimization process to refine the intensity distribution estimate as “variance shaping”. Simulations suggest that variance shaping indeed provides more accurate estimates than the simple SCORE at the cost of slight increase in computation time. Practically, for large image sizes, because the intensity fluctuation correlation between pixels is localized to single PSFs, large images are sliced into small overlapping regions, typically with a width of 6–10 times of the PSF size and processed independently in parallel. The individual regions are stitched together into the large image weighted by the total variance of each region.

### Quantitative evaluation of SCORE and comparison with STORM

The quality of single-emitter based methods such as STORM and PALM is determined by many factors including emitter brightness, switching duty cycle, and the number of frames that can be acquired. In addition, the localization precision for a single emitter scales with the width of the PSF and inversely with the square root of the number of photons received by the camera. Further reductions in precision occur in the presence of camera noise and ambient light collection. The on-off duty cycle of the emitters places an upper bound on the allowed emitter labeling density to achieve single emitter localization in a diffraction-limited area, which in turn defines the maximum spatial frequency of resolvable features according to the Nyquist criterion for a finite number of images collected. However, the actual density of localized emitters in the reconstructed image is proportional to the number of frames and the emitter duty cycle. It has been shown experimentally that image reconstruction quality can limited by either the localization precision or the emitter density, depending on the choice of the specific fluorophore [10]. In conventional single-frame based image reconstruction algorithms, the two factors independently limit the image quality. For instance, harvesting more photons in one switch-on event does not increase the allowed emitter density, and for a fixed emitter brightness, accumulating more frames does not improve localization precision. In contrast, by making use of the pixel intensity covariance, SCORE breaks the limits of the emitter density upper bound for each frame. Regardless of the amount of overlap between emitter PSFs, more switching events result in higher variance for a given emitter brightness and consequently higher signal-to-noise ratios (SNR) in the covariance matrix. Accumulating more frames provides better statistics of the pixel covariance, thus improving the ability to resolve smaller features.

**Figure 2:**
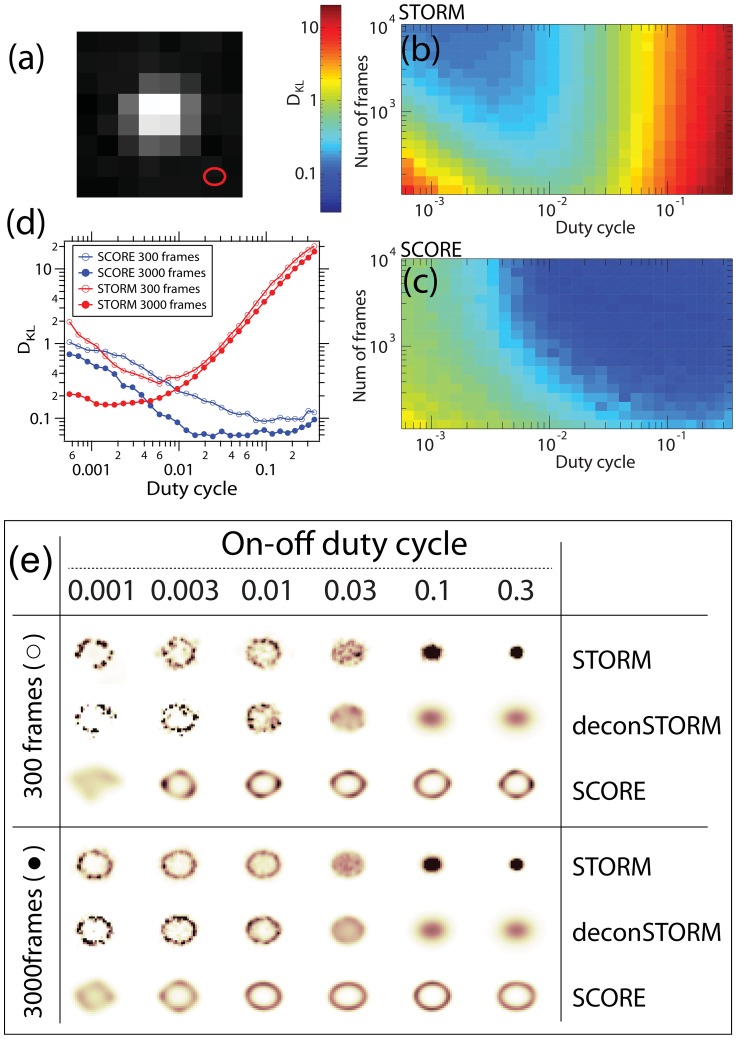
Comparisons of STORM and SCORE using simulated images from emitters evenly distributed on an sub-diffraction sized ellipse. (a) A Typical image of an emitter yielding 1000 photons on average, with an Gaussian-shaped PSF of width 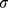 being 1 pixel. The size of the elliptical arrangement of emitters is shown at the bottom right in red. (b,c) The Kullback-Leibler (K-L) divergence of reconstructed STORM (b) and SCORE (c) images with the ground truth distribution at various duty cycle and number of frames in logarithm color scale. The values shown are the median of 40 repeats with identical parameters. (d) K-L divergence of two methods at stack size of 300 and 3000 frames as a function of duty cycle. (e) Sample images of STORM, deconSTORM and SCORE at various duty cycle and stack size.

To demonstrate the ability of SCORE to achieve super-resolution imaging using highly overlapping emitter PSFs, we first analyzed a series of simulated images. In the simulations, the width of the PSF, 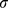, is set to 1 pixel, similar to typical cases where the pixel size is in the range of 100–150 nm (Fig. 2a). We place 100 emitters equally spaced on a sub-diffraction-sized ellipse with the long axis equal to 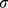 and the short axis equal to 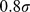 (red oval in Fig. 2a). The number of photons detected in each frame follows an exponential distribution with an average value of 1000 photons to simulate the variability of photons emitted during each switching event. Switching obeys two-state kinetics with a fixed off rate, 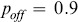, and on-rate, 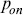. Gaussian white noise with a standard deviation of 5 is added to the simulated images to emulate read-out noise typical of EMCCD cameras. A set of reconstructed images using STORM and SCORE are then generated using values for 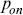 from 0.0005 to 0.5 and the total number of frames varying from 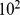 to 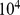.

To quantify the quality of the reconstructed images, we interpret each image as a probability distribution of emitter density, and use the Kullback-Leibler (KL) divergence to describe the distance between the ground truth and the images. The KL divergence is defined as
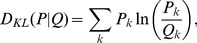
(8)where 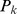 and 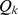 represent the emitter positions and the STORM or SCORE images respectively. Since the simulated emitters reside at a discrete set of locations, we convolve the emitter distribution with a Gaussian function whose width is equal to the size of an up-sampled grid spacing to reduce aliasing.

The KL divergences between STORM or SCORE images and the ground truth are shown in Figure 2b,c. Sample images reconstructed from 300 frames or 3000 frames under various duty cycles of the fluorophore switching are shown in Figure 2d along with a comparison between SCORE and STORM. For large numbers of frames (in the thousands) and low duty cycles (below a critical value of 0.01 where less than one of the 100 emitters is fluorescing at a time), STORM is able to reconstruct the ellipse with good accuracy, and SCORE has lower accuracy due to low emitter intensity variance at low duty cycles (Fig. 2e). Above the critical duty cycle, STORM quickly fails to resolve the hollow structure of the ellipse and the reconstructed image collapses to a single point. deconSTORM has a better tolerance to overlapping emitters but is still unable to resolve the ellipse if more than 3 emitters are active at a time (a duty cycle of 0.03). In contrast, SCORE consistently performs better at higher duty cycles and eventually reaches a KL divergence at 0.05, lower than the best performance of STORM at 0.2 for any duty cycle. The KL divergence of SCORE does not decrease further with duty cycle due to the anti-alias filtering of the ground truth and our automatic routine for determining the sharpness parameter 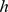. For a smaller number of images (300 frames), STORM and deconSTORM exhibit the same trend as the duty cycle changes when compared to the larger data set, but the reconstructed image quality is lower because of low SNR. In this regime, SCORE performs comparably to STORM, but the improvements of quality proceed monotonically beyond the critical duty cycle of 0.1, resulting in a consistently smoother and more accurate result (Fig. 2d,e).

**Figure 3:**
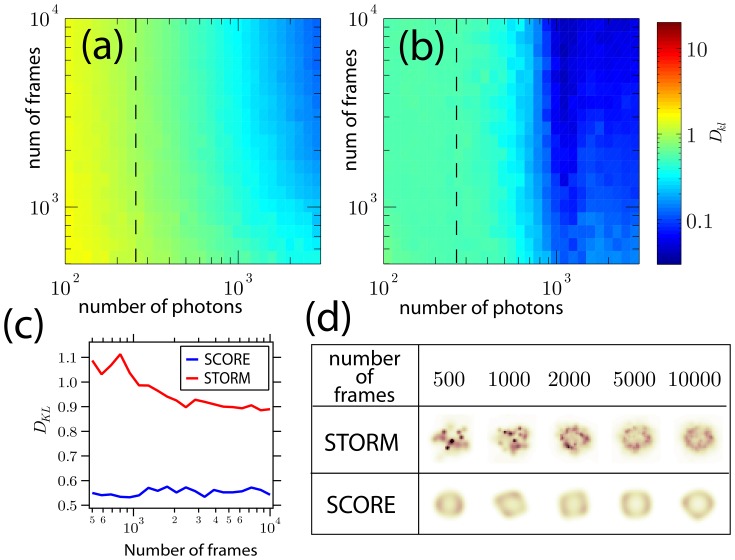
Comparison of STORM and SCORE at lower SNR where the number of photons per frame from each activated emitter is low and background noise is high. (a) The dependence of K-L divergence of STORM images on the number of accumulated frames and photons from each activated emitter per frame at high level of background noise (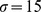). The values shown are the median of 40 repeats with identical parameters. (b) Same test as (a) on SCORE. (c) A slice of K-L divergence for STORM (red) and SCORE (blue) at 256 photons per frame, indicated as the dashed lines in (a) and (b). (d) Samples of reconstructed images from the two methods at 256 photons per frame and selected numbers of frames.

Another factor limiting the quality of the reconstructed image is the localization precision of each emitter, which is determined by its brightness and the level of background noise [22]. In single-emitter localization techniques, the uncertainty in determining emitter location effectively blurs the emitter positions and this can not be easily corrected by accumulating frames. To study the effect of localization precision on imaging quality, we increase the background noise in our simulation to a higher level, 15, and vary the SNR ratio by tuning the number of photons received from a emitter from 100 to 3000 per frame. The K-L divergence of STORM and SCORE images from the ground truth is calculated at their corresponding near-optimal duty cycle of 0.002 for STORM and 0.05 for SCORE (Fig. 3a,b). For a fixed number of frames, a higher number of photons increases the SNR, thus improving the reconstructed quality of both STORM and SCORE. To show the effect of accumulating frames, the K-L divergence when 256 photons per frame per emitter are collected is plotted in Figure 3c, and sample reconstructed images for various number of accumulated frames are shown in Figure 3d. Both in the K-L divergence and the sample images, SCORE produces consistently better quality images than STORM. However, STORM quality improves with an increasing number of images whereas SCORE quality is insensitive to this because determination of the sharpness parameter 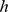 is conservative for high noise cases and thus limits the quality. One could include knowledge of the number of frames to adjust the sharpness parameter 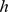 and improve the accuracy of SCORE.

### SCORE images of fluorescently labeled microtubules

As a demonstration, we applied SCORE imaging to fluorescentl microtubules labeled with an organic Alexa dyes and quantum dots (QDots). Microtubules from fixed HeLa cells were stained with anti-tubulin antibodies conjugated to the Alexa Fluor 647 fluorescent dye and imaged with epi-fluorescence microscopy. A 50 mW, 647 nm solid-state red laser was kept on during the entire image acquisition, yielding an estimated 1–10 kW/cm^2^. After a brief, few-second, exposure to the red laser, the majority of the fluorophores were switched off. We then added a millisecond laser pulse of 405-nm wavelength to stochastically switch on fluorescent molecules between camera acquisition cycles. After and initial 3–5 s burst where many molecules become activated, the duty cycle leveled of to a steady low value for minutes. Images were acquired at 100 frames per second to capture the fast dynamics of fluorophore switching.

**Figure 4:**
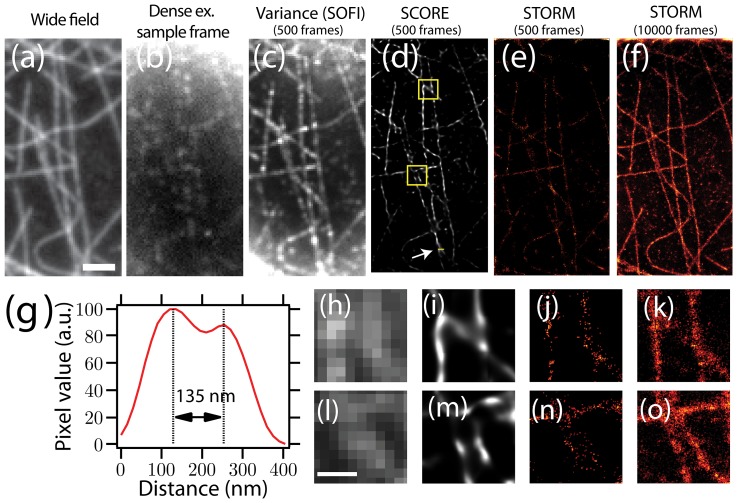
Microtubules in Hela cells labeled with Alexa Fluor 647 and analyzed with SCORE and STORM with various numbers of frames. (a) An epi-fluorescence image of labeled microtubules before the majority of the dye was switched off. (c) The variance of 500 frames where the 405 nm laser activated a high density of fluorophores with overlapping PSFs. (d) and (e) SCORE and STORM (using rapidSTORM) analysis of these 500 frames (5 seconds at 100 frames per second). (f) STORM analysis of 10,000 frames with a low density of switched-on emitters. (g) Intensity profile of a line in the SCORE image indicated by the arrow and yellow line in (d). (h)–(o) Two zoomed-in portions of the variance, SCORE, 500-frame STORM and 10,000-frame STORM indicated by the yellow box in (d). Scale bars: (a–e) 2 µm, (g–n) 500 nm.

We analyzed the first 5 seconds of data (from 500 frames) using SCORE after the UV activation laser was turned on, when the duty cycle was the highest (Fig. 4). From only 5 seconds of data, SCORE is able to resolve sub-diffraction limited separation of 135 nm between adjacent microtubules, where the Gaussian-approximated PSF width is 192 nm for deep red emission. For comparison, at the high duty cycle STORM analysis rejects many activated emitters, resulting in a low density of resolved centers (Fig. 4(e,j,n)). The next 10,000 frames had low duty cycle and are suitable for standard STORM analysis (Fig. 4f). Small details of the SCORE and STORM images do not exactly match because SCORE is sensitive to the blinking of the emitters, whereas STORM is sensitive to the presence of the emitters. The performance of organic dyes suffered from photobleaching after several switching cycles, and we optimized our conditions for STORM where intensity flickering is not fully promoted. It has been reported that mixing both oxidizing and reducing compounds with these dyes increases both switching-on and -off rates [23]. In these conditions, we anticipate the faster and more robust switching dynamics would yield better results using SCORE.

**Figure 5:**
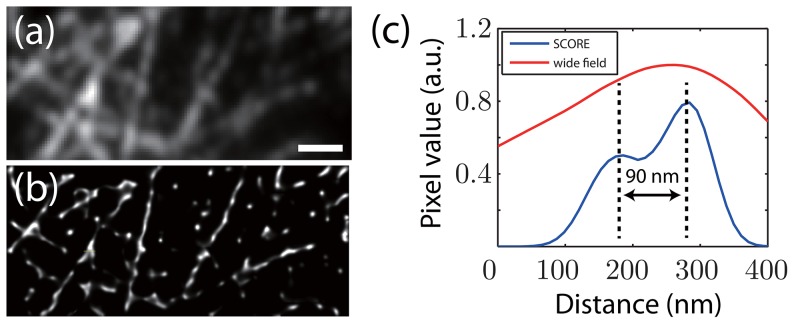
Quantum dot labeled microtubules in HeLa cells were imaged and analyzed with SCORE. (a) An averaged image of 1000 frames (7.4 seconds) of quantum dot labeled microtubules. (b) SCORE image of the same 1000 frames. STORM is not applicable due to the high density of QDots. (c) Intensity line profile of he labeled portion of the wide field image (indicated by the arrow in (b)) and SCORE image (blue line). The line profiles were obtained from linear interpolation of the original images. Scale bar: 1 µm.

Another type of fluorescent source that is bright and has intrinsic intensity fluctuations are QDots. In conventional imaging applications, the variability in brightness of QDots are often considered as negative factors. However, these intensity variations are crucial for fluctuation-based super-resolution techniques such as SCORE [18], [19]. We decorated microtubules in fixed HeLa cells with antibodies conjugated to QDots with an emission spectrum peaked at 655 nm. A 20 mW, 532 nm wavelength laser was used as the excitation source, and we imaged microtubules using oblique illumination with a 1.49 NA objective. The power-law distribution of the on- and off-times of QDots suggests that there is no typical time scale for intenstiy fluctuations [16], thus we chose to image at a fast frame rate of 135 frames per second. Compared to Alexa Fluor 647 labeled microtubules, QDot labeling was more heterogeneous and discontinuous along the microtubules (Fig. 5a), presumably due to the larger size of the QDots and our sample preparation quality. Figure 5a shows the averaged image of 1000 frames (7.4 seconds of acquisition), and the SCORE image is shown in panel b. The SCORE image clearly shows the gap between labeled microtubule segments (Fig. 5b), and is able to resolve a separation near the junctions of two crossing microtubules with better than 90 nm resolution (Fig. 5c).

## Discussion

Super-resolution imaging is now achievable on a conventional epi-fluorescence microscope with little to no modification to the optical design. The fine structural information embedded in a sequence of fluorescence images can be restored using single particle localization as implemented in STORM and PALM, or intensity fluctuation statistics as in SOFI, ICA and SCORE. STORM/PALM and related localization-based methods have become increasingly popular due to their outstanding spatial resolution. The quality of the reconstructed images are limited by essentially two factors: the localization precision, determined by the emitter brightness and background noise, and the sampling density, set by the fluorophore duty cycle, labeling density, and acquisition time. Either factor can be the bottleneck of the final image quality. Moreover, the duty cycle of the emitters is usually set much lower than the optimal value for experimentally practical reasons. Therefore, STORM/PALM typically require thousands of images to gain dense sampling due to the essential non-overlapping condition. In contrast, by looking at the covariance between the intensities of pixels statistically, SCORE is not limited by the non-overlapping condition, and is able to reconstruct images with comparable quality from far fewer frames that contain denser information. In addition, any improvements in emitter brightness and its variation, the number of frames acuired, higher labeling density, and lowered background noise contribute to better statistics, therefore the imaging quality is no longer limited by a single bottleneck. In particular, a higher labeling density is always preferred for SCORE. In the case of low emitter density, single particle localization methods make use of information that the statistical methods ignore, and are able to resolve probe locations with better accuracy at the cost of a substantially longer acquisition time.

SCORE is closely related, but different from existing statistical methods such as SOFI [17], [18] and ICA [19]. All three methods share the common advantages of analyzing intensity statistics over single particle localization methods as discussed above. SOFI and its later improvement XC-SOFI primarily use the temporal fluctuation of individual pixels. The spatial correlation (cross cumulant) information is used to interpolate estimation between pixels to achieve finer resolution. SCORE considers the covariance between all pairs of pixels to estimate the distribution of emitters, and by sorting and truncating the eigen modes, SCORE efficiently resolves the emitter distribution from a reduced linear subspace of principal components rather than the original image pixel space that has much higher dimension. SCORE and XC-SOFI share many common advantages over STORM/PALM, and have similar limitations as well [24]. Lidke *et al.* resolved the localization of a finite number of quantum dots from the temporal sequence of the eigen modes using ICA, however the ability to resolve individual emitters quickly becomes intractable if several emitters are located in one PSF area. Instead of attempting to resolve individual emitters with their explicit temporal blinking sequences as ICA does, our method focuses on the total variance and covariance derived from the ensemble average, and thus it is capable of estimating a continuous distribution, and is not limited to a small number of discrete points.

Because the covariance matrix does not depend on the basal intensity at each pixel, SCORE will reject any temporally non-drifting backgrounds such as an autofluorescence signal. This property allows SCORE to analyze images with only a portion of emitters fluctuating in a background of constantly fluorescing molecules. Because the fluorescence intermittence takes place at much faster time scale than the photobleaching, it is applicable to temporally high-pass filter the intensity sequence at each pixel to remove the slowly varying background signal before calculating the covariance. The out-of-focus light can be treated as background light since the intensity correlation of defocused light is also delocalized and spatially convolved into more uniform signals in time. Thus the contribution to the covariance becomes smaller as the emitter resides further from the focal plane.

Many different types of fluorescent sources undergo intensity fluctuations on fast time scales, and thus can serve as potential candidates for SCORE analysis. In this work, we used organic dyes and quantum dots to demonstrate SCORE imaging. Organic dyes can switch between bright and dark states in certain chemical environments, but they have a limited number of switching cycles before bleaching, and therefore limit the SCORE image [10]. For quantum dots, the high brightness, robust fluorescence intermittence and resistance to photobleaching are major advantages as fluorescent sources for SCORE. In the past decade, a library of fluorescent proteins have been reported to have stochastic or controllable intensity fluctuations or switchablility in emission spectra at fast time scales. These include mTFP0.7 [25], Dronpa [26], rsCherry [27], IrisFP [28], and rsTagRFP [29]. Dedecker *et al.* have successfully used SOFI to image Dronpa-labeled Lyn kinase [30] and resolved structure at 100–200 nm spatial scale. Their work demonstrated the feasibility to use fluorescent protein with statistical super-resolution methods like SCORE. Because the only two assumptions SCORE makes on the intensity fluctuation statistics are identity and independency, it is insensitive to the explicit fluctuation dynamics. This feature grants SCORE imaging great flexibility for use with a large variety of different fluorophores.

Even tough the computational complexity is often not considered as a major factor in choosing algorithm-based methods for super-resolution imaging, performance can become a practical limitation in certain circumstances. Because SCORE is based on analysis of the covariance of the entire image stack instead of a frame-by-frame analysis as in deconSTORM and CSSTORM, its speed is typically 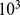 to 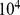 times faster than deconSTORM (Table 1). This performance is similar to STORM and PALM where simple Gaussian fitting is implemented and where the processing time for a 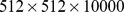 image stack is minutes. In practice, this improvement in speed allows one to iteratively optimize SCORE for the best image quality. Bayesian localization microscopy, on the other hand, achieves super-resolution in remarkably short time [21] but its heavy optimization in high-dimensional parameter space also requires significant computational resources such as cloud computing [31].

**Table 1:** Speed performances of various methods.

Method	Calculation time scale (s)
Variance	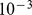
SCORE	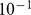
STORM	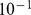
deconSTORM	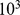
3B	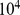

Typical time consumed by various methods to process a small sample image stack is listed for comparison. The sample consists of a stack of 16×16×500 images. The benchmark is performed on an Intel Core i5-650 CPU. Estimated time for the Bayesian localization microscopy (3B) is adopted from [34].

## Materials and Methods

### Cell Culture and Immunostaining

Human HeLa cells were cultured as described in [32]. Cells were fixed in formaldehyde followed by permeablization [32]. Fixed cells were washed in phosphate buffer saline (PBS) 3 times, incubated with mouse anti-tubulin (Sigma-Alderich) for 24 hours at 4°C, then washed with PBS 3 times, followed by an incubation in 20 nM anti-mouse IgG labeled with Alexa Fluor 647 (Life Technology) in PBS and 6% w/v BSA for 30 minutes. Stained cells were washed in PBS and replaced with imaging buffer containing 50 mM Tris at pH 8.0, 10 mM NaCl, 10% glucose w/v, 5 mg/ml glucose oxidase, 100 ug/ml catalase and 100 mM 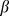
me [10]. All chemicals were purchased from Sigma-Alderich unless noted otherwise.

Quantum dot labeled HeLa cells were prepared and treated in the same way described above. Cells incubated with primary antibody were washed with PBS 3 times, and incubated with 20 nM anti-mouse IgG labeled with QDot 655 (Life Technology) in PBS and 6% w/v BSA for 30 minutes. Stained cells were washed in PBS and the slide was mounted on the microscope for imaging.

### Microscopy and Imaging

Alexa Fluor 647 labeled Microtubule *in vivo* images were taken on a home-built microscope with on Olympus 60× 1.4 NA oil-immersion objective. A 50 mW 647 nm solid-state laser (CrystaLaser) was used as the excitation and deactivation source, and a 100 mW 405 solid-state laser (Coherent) was used as the activation laser. Images were collected using an EMCCD camera (iXon+ 897E, Andor Technology). Quantum dot labeled microtubule images were taken on a Nikon TE-2000 inverted microscope. A home-built 20 mW 532 nm diode laser was used as the excitation source. The images were collected with an EMCCD camera (iXon+ 897E, Andor Technology) controlled by LabVIEW (National Instruments, Austin, TX).

### Image Analysis

For SCORE analysis, image stacks of Alexa Fluor 647 labeled microtubule were assumed to have a fixed background with a smoothly decaying intensity. The decaying background was estimated by fitting a single exponential as a function of time at each pixel, and then subtracted from the raw images before SCORE analysis. The image field is divided into overlapping regions of interest with size of 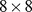 pixels, processed by SCORE, normalized by the variance of the ROIs, and stitched together with pyramidal weight masks (2D equivalent of triangle window function in 1D). The square masks are tiled with a small overlap so that the corners reside on the centers of the other set of square masks. Quantum dot images did not have observable photobleaching, thus background correction was not necessary. The whole image was divided into 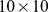 overlapping regions of interest and stitched in the same way described above.

STORM images in the simulations were reconstructed from the Gaussian fit of the individual frames, since all emitters were located with 1 PSF width, no separate or Gaussian mixture fit was possible or necessary. The STORM images were generated and rendered from the localization results, and each detected emitter is displayed as a 2D Gaussian function whose width scales with the inverse square root of the emitter intensity as described in [10]. Images of Alexa dye was processed and the STORM images were rendered using rapidSTORM [33]. All simulations, Gaussian fit in STORM simulations, and STORM image rendering and SCORE analysis were performed in MATLAB (The MathWorks, Matick, MA).

## Supporting Information

File S1 **Supporting material.**
(PDF)

Figure S1 **The principle of variance optimization in SCORE.** (a) The first 10 eigen modes of a 8×8 pixel portion of 500 frames from experimental data. (b) The variance of the 500 frames (upper panel) and the SCORE image (lower panel). (c) The variances in the basis of eigen modes (diagonal elements of the covariance matrix). The red squares are the experimental variances, the blue line is the fit using a double-exponential function, and the green solid circles are the calculated variances from the reconstructed image. (d) The calculated covariance matrix of the reconstructed image. The covariance matrix of the experimental data is a diagonal matrix with diagonal elements shown in (c).
(EPS)
