# Supplementary material for: Spatial Covariance Reconstructive (SCORE) Super-Resolution Fluorescence Microscopy
Source: PLoS One. 2014 Apr 30;9(4):e94807. doi: 10.1371/journal.pone.0094807 (PMC4005777; doi:10.1371/journal.pone.0094807)
Supplement: File S1 — Supporting material. (PDF) [file pone.0094807.s001.pdf]

Supporting Material for Spatial Covariance Reconstructive (SCORE) Super-Resolution Fluorescence Microscopy

Yi Deng Mingzhai Sun Peihui Lin Jianjie Ma
Joshua W. Shaevitz*

Calculate covariance from any emitter distribution

Given an emitter distribution n_j on the refined grid $\{x_j\}$, assuming the emitter intensity fluctuation statistics is independent, identical and normalized, the covariance matrix under an orthonormal basis g_l can be calculated. Let $s_{m,t}$ be the temporal sequence of the mth emitter at time point t, with $m = 1, 2, \ldots, M$ and $t = 1, 2, \ldots, T$, j_m the position index of the mth emitter, and f_j the PSF centered at the jth pixel in the refined grid, the image at time t is

$$I_{j,t} = \sum_m f_{j_m} s_{m,t}. \tag{S1}$$

The components on g_l is

$$\begin{aligned} c_{l,t} &= \langle g_l, I_{j,t} \rangle && \text{(S2)} \\ &= \sum_m \langle g_l, f_{j_m} \rangle s_{m,t} && \text{(S3)} \\ &= \sum_m A_{l j_m} s_{m,t}, && \text{(S4)} \end{aligned}$$

*Email: shaevitz@princeton.edu

and the covariance is

$$
\begin{aligned}
C_{ll'} &= E\{c_{l,t}c_{l',t}\}_t && \text{(S5)}\\
&= \sum_{m,m'} A_{lj_m}A_{l'j_{m'}}E\{s_{m,t}s_{m',t}\} && \text{(S6)}\\
&= \sum_{m} A_{lj_m}A_{l'j_m} && \text{(S7)}\\
&= \sum_{j} A_{lj}A_{l'j}n_j, && \text{(S8)}
\end{aligned}
$$

because of the independence and normalization of the fluctuation sequence

$$E\{s_{m,t}s_{m',t}\} = \delta_{m,m'}, \tag{S9}$$

and

$$\sum_{j_m=j} 1 = n_j. \tag{S10}$$

Optimizing the variance (variance shaping)

Let $C_{ii'}$ be the intensity covariance between the ith and the i'^{th} pixel. $C_{ii'}$ is diagonalized to $C_{jj'}$ by the eigen vectors of $C_{ii'}$, with the diagonal elements the eigen values. Fig. S1(a) shows the the first 10 eigen modes of a 8×8 pixel portion of 500 frames from experimental data, and the variances of the total 64 eigen modes are sorted and plotted in Fig. S1(c) as red open squares. The variance as a function of the mode index is then fit with a double exponential function:

$$V(l) = \alpha_1 e^{-l/\beta_1} + \alpha_2 e^{-l/\beta_2}, \tag{S11}$$

where l is the mode index, and $\alpha_1 > \alpha_2$. The fitting result is shown as blue line in Fig. S1(c). The location of l_0 is indicated by the dashed line. In all of our experimental data and simulations, the two exponential function intersect at l_0 that is positive:

$$l_0 = \frac{\beta_1\beta_2}{\beta_1 - \beta_2}\ln\frac{\alpha_1}{\alpha_2}. \tag{S12}$$

We truncate the number of modes in the variance optimization according to l_0, in this work we keep extra 2 modes to better recover the details of the

details in the reconstructed images. From simulations and experimental data, we found that including an extra 10% of the total models to the optimization does not improve or degrade the reconstruction quality, but only increase the time the optimization takes to converge.

The sharpness parameter h is also derived from the double exponential fit to the variances. Because h determines the gradient of the reconstructed image, and the resolution is intrinsically limited by the signal to noise ratio (SNR), h can be imperially written as a function of the SNR, which is estimated from $\alpha_1, \alpha_2, \beta_1$ and β_2:

$$\max(|\nabla n_j|) = \left[(0.278e^{-(\alpha_1/\alpha_2)/455} + 0.083)\sigma\right]^{-1}, \tag{S13}$$

where σ is the width in pixels of the PSF in the reconstructed image, and the sharpness factor is numerically solved from the desired magnitude of the gradient scaled by the PSF size, with a scaling factor depending on the SNR α_1/α_2. In order to manually turn the sharpness of the reconstructed image, one can multiply an additional factor to the right hand side of the equation above.

Symbol definition

Table S1 Symbols that are used in the main text and the supplementary materials.

i	Pixel index of the observed image
j	Pixel index of the refined reconstructed image
x_j	Location of the jth pixel in the refined grid
l	Eigen mode index
l_0	Index of the last significant eigen mode
m	Emitter index
t	Time index
n_j	Number of emitter on the jth refined grid position
j_m	Position index of the $m^{\textbf{th}}$ emitter on the refined grid
$c_l(t)$	Coefficient of the lth eigen mode of an image at time t
$C_{ll'}$	The covariance matrix in the basis of eigen modes
f_j	Gaussian PSF centered at the jth pixel in the refined grid
g_l	l^{th} eigen mode
D_j	Distance of f_j to the subset of significant eigen modes
r_l	Variance profiling parameters
A_{lj}	Projection of PSF f_j on eigen mode g_l
λ_l	The variance of the lth eigen mode
$w_{ll'}$	Weighting parameter in variance shaping optimization
h	Sharpness parameter
σ	Gaussian PSF width
$\langle , \rangle$	Inner product
$E\{\}_t$	Average over index t

Supporting Information Legends

Fig. S1 The principle of variance optimization in SCORE. (a) The first 10 eigen modes of a 8×8 pixel portion of 500 frames from experimental data. (b) The variance of the 500 frames (upper panel) and the SCORE image (lower panel). (c) The variances in the basis of eigen modes (diagonal elements of the covariance matrix). The red squares are the experimental variances, the blue line is the fit using a double-exponential function, and the green solid circles are the calculated variances from the reconstructed image. (d) The calculated covariance matrix of the reconstructed image. The covariance matrix of the experimental data is a diagonal matrix with diagonal elements shown in (c).
